# Hospital performance and payment: impact of integrating pay-for-performance on healthcare effectiveness in Lebanon

**DOI:** 10.12688/wellcomeopenres.15810.2

**Published:** 2020-12-10

**Authors:** Jade Khalife, Walid Ammar, Maria Emmelin, Fadi El-Jardali, Bjorn Ekman

**Affiliations:** 1 Faculty of Medicine at Lund University, Lund, Sweden; 2 Ministry of Public Health, Beirut, Lebanon; 3 Faculty of Health Sciences, American University of Beirut, Beirut, Lebanon

**Keywords:** performance, health systems, reform, casemix index, low and middle income countries, interrupted time series analysis, unnecessary hospitalization, coding

## Abstract

**Background**: In 2014 the Lebanese Ministry of Public Health integrated pay-for-performance into setting hospital reimbursement tiers, to provide hospitalization service coverage for the majority of the Lebanese population. This policy was intended to improve effectiveness by decreasing unnecessary hospitalizations, and improve fairness by including risk-adjustment in setting hospital performance scores.

**Methods**: We applied a systematic approach to assess the impact of the new policy on hospital performance. The main impact measure was a national casemix index, calculated across 2011-2016 using medical discharge and surgical procedure codes. A single-group interrupted time series analysis model with Newey ordinary least squares regression was estimated, including adjustment for seasonality, and stratified by case type. Code-level analysis was used to attribute and explain changes in casemix index due to specific diagnoses and procedures.

**Results**: Our final model included 1,353,025 cases across 146 hospitals with a post-intervention lag-time of two months and seasonality adjustment. Among medical cases the intervention resulted in a positive casemix index trend of 0.11% per month (coefficient 0.002, CI 0.001-0.003), and a level increase of 2.25% (coefficient 0.022, CI 0.005-0.039). Trend changes were attributed to decreased cases of diarrhea and gastroenteritis, abdominal and pelvic pain, essential hypertension and fever of unknown origin. A shift from medium to short-stay cases for specific diagnoses was also detected. Level changes were attributed to improved coding practices, particularly for breast cancer, leukemia and chemotherapy. No impact on surgical casemix index was found.

**Conclusions**: The 2014 policy resulted in increased healthcare effectiveness, by increasing the casemix index of hospitals contracted by the Ministry. This increase was mainly attributed to decreased unnecessary hospitalizations and was accompanied by improved medical discharge coding practices. Integration of pay-for-performance within a healthcare system may contribute to improving effectiveness. Effective hospital regulation can be achieved through systematic collection and analysis of routine data.

## Introduction

The linkage of performance and payment has been increasingly used in healthcare during the past two decades. In recent years this has extended towards hospital performance designs, despite mixed evidence regarding its effects.

In 2014 the Lebanese Ministry of Public Health (MoPH) changed the basis by which it determined the payment reimbursement tiers of about 140 public and private hospitals, which it contracts for providing hospitalization service coverage for the majority of the Lebanese population. A pay-for-performance (P4P) framework was integrated within this system, the most prominent component of which was the hospital casemix index (CMI), which reflects the average risk or illness severity of all patients within a hospital ( Khalife *et al*., 2017). This intervention was aimed at improving effectiveness by incentivizing hospitals to decrease unnecessary hospitalizations, as well as to improve fairness in determining hospital reimbursement (by including risk-adjustment), within an integrated evaluation framework.

Hospitalizations that are not compliant with any medical or social criteria may be considered as unnecessary hospitalizations. These arguably differ from potentially preventable hospitalizations; in that the latter may have an indication for admission but would have otherwise been avoided with appropriate outpatient or primary care. Unnecessary hospitalizations are common in various countries and challenge the functioning of healthcare systems ( Caminiti *et al*., 2013; Macinko *et al*., 2010; Stranges & Friedman, 2006; To *et al*., 1996). Such cases may be primarily regarded as a question of appropriateness of care. Using the Kruk and Freedman performance framework, unnecessary hospitalizations may be categorized as a quality of care output measure, under the effectiveness dimension ( Kruk & Freedman, 2008). Downstream association with outcomes on patient health status (effectiveness) and on maximizing value of resources (efficiency) would also be expected.

The current evidence on the impact of P4P in healthcare is weak, particularly in low- and middle-income countries (LMICs) ( Witter *et al*., 2012). In this study we assess the impact of the integration of the P4P policy by the MoPH, and contribute to the evidence base on P4P effectiveness, using routine data and a specially developed CMI.

### Pay for performance

A health reform that links measures to payment creates a financial incentive for service providers to improve their performance vis-à-vis these measures. Seen through the lens of principal-agent theory, such P4P seeks to address the recognized information problems within healthcare, particularly information asymmetry ( Smith & Hanson, 2012). This alignment of interests provides the principal (i.e. the payer) a tool to incentivize the provider to improve healthcare outputs and outcomes ( Grace *et al*., 2015). Such tools may function at a system level within a complex environment and should be adjustable by the principal ( Roberts *et al*., 2004). Aspects of a P4P intervention that have a large role in determining impact include the actual measures used, context and incentive size.

Most performance frameworks have either been developed for use in high-income countries (HICs) or are heavily influenced by such contexts, and likely require adjustment for use in LMICs ( Tashobya *et al*., 2014). Based on a review of commonly used performance indicators, Kruk and Freedman provide a framework for LMICs ( Kruk & Freedman, 2008). Adapting Donabedian’s system evaluation of structure, process and outcome measures, this framework categorized indicators as outputs/processes and outcomes/impact along with the dimensions of effectiveness, equity and efficiency ( Donabedian, 1966; Donabedian, 1988).

In LMICs, healthcare P4P initiatives commonly include structural measures of quality. More widely, however, a transition is ongoing towards outcome measures, which are the ultimate target for performance improvement ( Chee *et al*., 2016; Gergen *et al*., 2017). Establishing broad outcome measurements may enhance P4P impact and integration into routine systems may make such initiatives more cost-effective ( Borghi *et al*., 2015; Chee *et al*., 2016).

The effects of healthcare P4P in HIC contexts have been mixed, largely confirming the scarcity of the evidence base ( Eijkenaar *et al*., 2013; Emmert *et al*., 2012; Gillam *et al*., 2012; Van Herck *et al*., 2010). A recent review on P4P schemes in the United Kingdom found some positive findings, but cautioned that overall effects on care quality were unclear, underscoring the need for long-term monitoring and evaluation ( Mandavia *et al*., 2017).

The potential for P4P impact may be larger in LMICs, considering the relatively lower resources of providers and more dynamic health reform context ( Witter *et al*., 2012). However, the scarcity of the evidence from LMICs is particularly pronounced; the evidence base is too weak to make general conclusions ( Witter *et al*., 2012). A recent review of P4P in maternal and child care in LMICs found positive impact on process quality, but weak evidence on health outcomes and out-of-pocket expenses ( Das *et al*., 2016).

Hospital-based P4P initiatives in England and the United States targeting 30-day readmissions and mortality resulted in improved short-term performance that was not sustained in the long-term, further highlighting challenges due to contextual changes, spill-over, measurement limitations and overall design ( Jha *et al*., 2012; Kristensen *et al*., 2014; Lindenauer *et al*., 2007; Sutton *et al*., 2012; Werner *et al*., 2011). Long-term investigations of previously favorable initiatives have found improvement in readmissions, but no improvement or worsening of mortality, as well as undesirable practices having misled some earlier findings ( Gupta & Fonarow, 2018; Wadhera *et al*., 2018; Wasfy *et al*., 2017). Anticipatory or short-term behaviors in response to P4P scheme engagement and implementation may differ from long-term behavior, likely contributing to the findings of short but not long-term effectiveness of P4P ( Ryan *et al*., 2015).

It has been proposed that the debate on P4P should move from distinct projects towards integration within the health system, with broad system objectives ( Soucat *et al*., 2017). Such integration may be seen as an extension of strategic purchasing of health services, working towards achieving universal health coverage. An approach that considers the overall health system rather than more narrow objectives would avoid ‘not seeing the forest for the trees’ ( Soucat *et al*., 2017).

### Aim and objectives

We apply a systematic approach to assess whether the new pay-for-performance policy had an impact on the healthcare system’s effectiveness. We analyze whether this policy affected the complexity of the average hospitalization case (i.e. CMI), stratifying by hospitalization case types, length of stay, and hospital ownership. We further quantify any changes, with plausible explanations, at the level of diagnoses and procedures.

The specific objectives are:

1. To determine the impact on CMI level and trend, across public and private hospitals
  a. by case types: medical, surgical and mixed
  b. by length of stay: medical short, medium and long-stays

2. To detect any changes in diagnoses and procedures
  a. by contribution to CMI changes
  b. by hospitalized cases

### The Lebanese health care context and reforms

Lebanon is a small Mediterranean country of 4.5 million citizens and 2 million refugees (primarily Syrian) ( United Nations, 2017). The diversified healthcare system is dominated by public payers and private providers ( Ammar, 2003). The MoPH is the largest public payer, covering hospitalization for about 52% of Lebanese, who otherwise lack any insurance coverage for hospitalization ( Ammar, 2009). This role is a legacy of the 1975–1990 war that disrupted developmental reforms at various stages.

The MoPH has engaged in different healthcare supply-side reforms. Contracting private hospitals to provide hospitalization services took place in the 1960s due to public coverage limitations and political reasons, preceding neoliberal influences that supported New Public Management agendas in other systems ( Smith & Hanson, 2012). In the late 1990s public hospitals were granted semi-autonomous status, similar to contemporary experiences of public hospitals in HICs and LMICs.

Hospital accreditation was linked to payment in 2001 and incentivized hospitals towards quality improvement, with accreditation results used to categorize hospitals across three reimbursement tiers ( Ammar *et al*., 2007). Hospital budgets set on an annual basis were not targeted by this change, as they continued to be historically and politically determined. Therefore, a moral hazard existed for hospitals interested in admitting more predictable low-risk patients to efficiently reach their pre-allocated budgets. Unnecessary hospitalizations became increasingly recognized as a major problem, particularly for medical (non-surgical) cases where lower barriers to treatment existed ( Kronfol *et al*., 2014). These were often one or two-day stays with variable costs but generally less complex diagnoses or symptoms, such as nausea, vomiting, diarrhea and gastroenteritis ( Kronfol *et al*., 2014).

### Policy reform

To address unnecessary hospitalizations and increase fairness in hospital performance assessment, the MoPH created a new policy intervention in late 2014, linking reimbursement tier to a composite hospital total performance score (TPS) ( Ammar *et al*., 2013). The first public announcement of the intervention was through an engagement event held in August 2014 for hospital executives and managers. The event highlighted the new model and its components, as well as the importance of accurate coding for appropriate casemix assessment. The TPS included components on CMI, patient satisfaction and other minor policy indicators, as well as accreditation ( Khalife *et al*., 2017). Specifically, this policy had a regulatory aspect in the form of accreditation, and more distinct payment aspect for other components. The CMI reflects the average risk or illness severity of all patients within a hospital, and was effectively the greatest determinant of a hospital’s TPS ( Hornbrook, 1982). Therefore, a decrease in unnecessary hospitalizations would be expected to increase a hospital’s CMI and consequently its TPS. Additional information regarding this process has been described elsewhere ( Khalife *et al*., 2017).

The incentive size for tier classification remained unchanged from pre- to post-intervention periods. Broadly, the difference in reimbursement tier is 10% additional change per tier among surgical procedures, and about 15% per tier among medical cases; e.g. a bottom-tier ‘T3’ hospital charges 1,000,000 LBP (USD 670) for a surgical procedure, while a top-tier ‘T1’ hospital charges 1,200,000 (USD 800) for the same procedure.

Consequently, the policy intervention was directed at the mechanism whereby hospitals were categorized into the different reimbursement tiers. However, the intervention did not change the reimbursement structure of hospitalization cases. Reimbursement of medical cases remained fee-for-service, while surgical cases used a pre-defined flat-fee.

## Methods

### Ethical statement

Research protocol approval was granted by the Institutional Review Board (IRB) at the American University of Beirut (ID: FHS.FE.21). The requirement for patient consent was waived by the IRB.

### Data sources

Hospitalization data including all cases under MoPH coverage from January 2011 to December 2016 was extracted by the MoPH Information Technologies Department and shared with the research team in a format with patient identifiers anonymized. The fields included record number (unique per admission), case identifier, hospital code, admission date, discharge date, length of stay, total charge, medical code on each admission and discharge, and surgical procedure code. STATA software package version 11 was used for all calculations and analyses.

### CMI calculation

The MoPH overcame limitations in developing a hospital CMI for its contracted 146 public and private hospitals, due to the lack of local Diagnosis Related Groups (DRGs) on which most casemix systems rely on. The MoPH CMI calculation approach used average costs for weight-setting among medical cases based on International Classification of Diseases, 10th Revision (ICD-10) discharge code, and Common Procedural Terminology (CPT) procedure code among surgical cases ( Ammar *et al*., 2013; World Health Organization, 2006; Yang & Reinke, 2006). Among medical cases the weight-setting process was separated across short-stay (<2 days), medium-stay (2–15 days) and long-stay cases (>15 days). This approach has been detailed elsewhere ( Ammar *et al*., 2013; Khalife *et al*., 2017).

For greater comparability across case types, we standardized the weight-setting previously used by the MoPH; we used five-year cost averages rather than the MoPH two-year averages; and assigned the average weight among medical cases for low-volume medical conditions (less than 20 cases). Surgical CMI did not require average cost figures as surgical services have fixed flat-rate charges. However, 11 procedures with weights ten times above the standard reference (1 million LBP) were capped at a weight of 10.00 to limit excessive impact of outliers.

The weight for each ICD and procedure code was the same throughout the period investigated. We note that the MoPH updated procedure costs in March 2013, which remained in use until the 2018 update. This increased the base-rate of all procedures, to account for inflation. We used weights based on the March 2013 update, also retrospectively up to 2011. The MoPH undertook a hospital-bed update (base-rate), which is only one component of the bill charged by hospitals to the MoPH, but nevertheless represents an internal inflation adjustment. For medical cases, we used the five-year code average without further adjustment.

We developed algorithms to calculate monthly CMI (rather than yearly) and developed ‘mixed’ cases algorithms (cases concurrently including medical and surgical care). We incorporated secondary procedures into surgical CMI, but this was not done for medical CMI as additional diagnoses or comorbidities are not currently utilized.

CMI was calculated using the formula below, which excludes the denominator correction component used in some versions, as this was calculated at system rather than hospital level ( Lichtig, 1986). This is also the standard generic CMI formula used by the US Centers for Medicare and Medicaid Services ( Services, 2011).

$$\text{CMI}=\frac{\sum \left[W_{g}*N_{gn}\right]}{\sum_{g}N_{gn}}$$

where W _g_ is the weight calculated for each ICD or CPT, and N _gn_ is the number of cases within each ICD in the total population.

CMI was calculated for medical, surgical and mixed cases separately, and repeated using cases only at public hospitals and only at private hospitals separately. Among the medical cases, short-stay, medium-stay and long-stay casemix indices were also calculated separately at each stage and combined using a case-weighed approach to also obtain an all-stay medical CMI. Such a combination was not conducted for all cases combined as it would likely conceal meaningful findings.

We excluded all chemotherapy coded cases (ICD Z51.1 and Z51.2). Chemotherapy is generally a low-cost short-stay hospitalization, and the MoPH has since 2014 communicated to hospitals the need for correct coding specifically for cancer patients. As a result, the MoPH has documented a decrease in chemotherapy miscoding under general cancer codes (e.g. C50 code being corrected as Z51.1). Retaining chemotherapy codes would have had the effect of artificially decreasing CMI. Typically, chemotherapy cases form around 7.9% of medical cases or 4.4% of total cases.

CMI algorithms were first run on an annual level for developing descriptive statistics. Similar algorithms were used to develop a monthly CMI with discharge date used to categorize records into calendar months.

### Study design and methods

***Impact on CMI level and trend, across public and private hospitals (Objective 1).*** To detect a change in CMI attributable to our intervention, it is necessary to make use of a control, whether outside of the group (using randomization) or within it (using historical control), while accounting for potential confounders. Randomization was not possible in this situation due to legal regulations that necessitate the MoPH to use the same performance assessment and reimbursement framework for all acute-care hospitals. Considering the availability of multi-year data, we chose to use a single-group interrupted time series (ITS), with Newey ordinary least squares regression. ITS analysis is considered the most appropriate quasi-experimental design, given the research context and aims ( Penfold & Zhang, 2013). With such an approach we seek to identify an ‘interruption’ of a continuous sequence of observations in a population (a time series) by a specific intervention. ITS analysis is particularly useful when randomization is not possible, and may have greater external validity than randomized designs when occurring in a real-world setting ( Bernal *et al*., 2016). This approach uses a historical control group, and more broadly the Bradford Hill criteria for causality to offer plausible causal explanations ( Habicht *et al*., 1999; World Health Organization, 2009; World Health Organization, 2012). Such an approach has also been noted as the most suitable for the evaluation of P4P interventions ( World Health Organization, 2009).

ITS analysis has been increasingly used in population-level evaluations of health interventions, with advantages including the control for secular trends, ease of population-level analysis and ability to evaluate intended and unintended outcomes ( Bernal *et al*., 2016; Lopez Bernal *et al*., 2018). It also allows us to evaluate both the short- and long-term impact of an intervention, by analysis of level and trend changes, respectively, and when appropriately used allows us to limit the effects of history bias in what is a complex real-world setting ( Naci & Soumerai, 2016).

We applied ITS analysis on a dataset including all cases between January 2011 to December 2016, with the exception of the mixed cases dataset that began January 2012 (see Table 1). Statistical significance was set at p < 0.05. It is relevant to note that discharge codes were recorded in the hospitalization database as of January 2011 for most hospitals, with a few hospitals reaching full compliance within five months. Mixed cases data was available as of January 2012.

**Table 1.** Hospitalization cases under the Ministry of Public Health coverage at public and private hospitals in Lebanon, 2011–2016.

**All hospitals**	Case type		2011		2012		2013		2014		2015		2016		All years	
			n	%	n	%	n	%	n	%	n	%	n	%	n	%
	**Medical**	**Short-stay**	19,574	10.4	26,044	11.5	27,119	11.5	26,720	11.3	24,578	10.4	24,220	10.5	148,310	11.0
		**Medium-stay**	79,264	42.3	94,523	41.8	101,132	43.0	101,524	42.9	107,379	45.3	101,772	44.2	585,809	43.3
		**Long-stay**	2,156	1.1	2,851	1.3	2,793	1.2	2,770	1.2	2,674	1.1	2,494	1.1	15,744	1.2
		***All medical cases***	***100,994***	***53.8***	***123,418***	***54.6***	***131,044***	***55.8***	***131,014***	***55.4***	***134,631***	***56.8***	***128,486***	***55.7***	***749,863***	***55.4***
	**Surgical**	**Procedures**	93,516	-	111,355	-	112,523	-	114,021	-	110,168	-	109,083	-	650,666	-
		***Cases***	***83,025***	***44.3***	***98,396***	***43.6***	***99,767***	***42.5***	***100,934***	***42.7***	***97,037***	***40.9***	***95,602***	***41.5***	***574,975***	***42.5***
	**Mixed**	**Procedures**	5,247	-	6,358	-	6,164	-	6,668	-	8,241	-	8,932	-	41,610	-
		***Cases***	***3,584***	***1.9***	***4,120***	***1.8***	***4,114***	***1.8***	***4,446***	***1.9***	***5,517***	***2.3***	***6,396***	***2.8***	***28,187***	***2.1***
	**Total** **cases**		**187,603**	**100**	**225,934**	**100**	**234,925**	**100**	**236,394**	**100**	**237,185**	**100**	**230,484**	**100**	**1,353,025**	**100**
**Public**	Case type		2011		2012		2013		2014		2015		2016		All years	
			n	%	n	%	n	%	n	%	n	%	n	%	n	%
	**Medical**	**Short-stay**	6,301	12.7	9,263	13.3	9,178	13.0	9,541	12.8	10,051	12.8	10,634	12.4	55,033	12.8
		**Medium-stay**	20,968	42.2	29,229	41.8	30,672	43.3	33,680	45.3	37,492	47.8	40,832	47.7	193,094	45.0
		**Long-stay**	501	1.0	747	1.1	633	0.9	630	0.8	669	0.9	704	0.8	3,889	0.9
		***All medical cases***	***27,770***	***56.0***	***39,239***	***56.2***	***40,483***	***57.2***	***43,851***	***59.0***	***48,212***	***61.5***	***52,170***	***60.9***	***252,015***	***58.7***
	**Surgical**	**Procedures**	24,049	-	33,463	-	33,031	-	33,055	-	32,871	-	36,471	-	192,940	-
		***Cases***	***20,906***	***42.1***	***29,167***	***41.8***	***28,845***	***40.7***	***28,896***	***38.9***	***28,485***	***36.3***	***31,491***	***36.8***	***167,990***	***39.1***
	**Mixed**	**Procedures**	1,476	-	2,430	-	2,275	-	2,367	-	2,423	-	2,726	-	13,697	-
		***Cases***	***957***	***1.9***	***1,453***	***2.1***	***1,492***	***2.1***	***1,593***	***2.1***	***1,678***	***2.1***	***1,997***	***2.3***	***9,180***	***2.1***
	**Total** **cases**		**49,633**	** 100.0**	**69,859**	** 100.0**	**70,820**	** 100.0**	**74,340**	** 100.0**	**78,375**	** 100.0**	**85,658**	** 100.0**	**429,185**	** 100.0**
**Private**	Case type		2011		2012		2013		2014		2015		2016		All years	
			n	%	n	%	n	%	n	%	n	%	n	%	n	%
	**Medical**	**Short-stay**	13,273	9.6	16,781	10.8	17,941	10.9	17,179	10.6	14,527	9.1	13,586	9.4	93,287	10.1
		**Medium-stay**	58,296	42.3	65,294	41.8	70,460	42.9	67,844	41.9	69,887	44.0	60,940	42.2	392,721	42.5
		**Long-stay**	1,655	1.2	2,104	1.3	2,160	1.3	2,140	1.3	2,005	1.3	1,790	1.2	11,854	1.3
		***All medical cases***	***73,224***	***53.1***	***84,179***	***53.9***	***90,561***	***55.2***	***87,163***	***53.8***	***86,419***	***54.4***	***76,316***	***52.8***	***497,862***	***53.9***
	**Surgical**	**Procedures**	69,467	-	77,892	-	79,492	-	80,966	-	77,297	-	72,612	-	457,726	-
		***Cases***	***62,119***	***45.0***	***69,229***	***44.4***	***70,922***	***43.2***	***72,038***	***44.5***	***68,552***	***43.2***	***64,111***	***44.4***	***406,971***	***44.1***
	**Mixed**	**Procedures**	3,771	-	3,928	-	3,889	-	4,301	-	5,818	-	6,206	-	27,913	-
		***Cases***	***2,627***	***1.9***	***2,667***	***1.7***	***2,622***	***1.6***	***2,853***	***1.8***	***3,839***	***2.4***	***4,058***	***2.8***	***18,666***	***2.0***
	**Total** **cases**		**137,970**	**100.0**	**156,075**	**100.0**	**164,105**	**100.0**	**162,054**	**100.0**	**158,810**	**100.0**	**144,485**	**100.0**	**923,499**	**100.0**

The intervention was given a two-month lag for expected effect (i.e. as of October 2014), based on the MoPH experience of the hospital response time (personal communication; Dr. Jihad Makouk, MoPH). We adjusted for seasonality using calendar months. Using 72 monthly data points, and CMI as the dependent variable, we ran ITS separately for each of medical all-stay, short-stay, medium-stay and long-stay cases; surgical cases and mixed cases. We subsequently stratified into public and private hospital cases.

As part of the sensitivity analysis we also varied lag period between intervention and impact by zero to four months, with the overall results being unchanged except in magnitude. To ensure the adequacy of our ITS model, we assessed auto-correlation using the Cumby-Huizinga test; in most cases auto-correlation was not present, and where detected was otherwise accounted for by the lag period.

***Changes in diagnoses and procedures (Objective 2).*** We used a before and after approach to quantify the change in individual diagnoses and procedures in terms of CMI change (contribution to changes detected in Objective 1), and in terms of absolute and relative change to all hospitalizations. We used algorithms on a dataset including all medical and surgical cases, with a pre-intervention period combining 2013 and 2014 cases and a post-intervention period with 2015 and 2016 cases. This objective used annual cut-offs, comparing 2013 and 2014 cases with 2015 and 2016 cases, in contrast to the first objective (monthly). This was to allow for the development of policy-relevant information and allow CMI algorithm looping and comparison across around 10,000 ICD and CPT codes.

For each case type and stay, we selected the top twenty codes with the greatest CMI change from pre- to post-intervention periods, for code-level analysis. We therefore restricted our analysis to examine the codes with the greatest impact on CMI in any direction (i.e. positive or negative).

In our approach, the codes which had the greatest *impact* on overall CMI are identified as a function of code weight in relation to the overall CMI ‘average’, the code weight itself, and frequency. While the codes with the greatest change in terms of their *share* of CMI are identified as a function of code weight and frequency only.

CMI change formulae:

$${\text{WN}}_{0}={\left({\text{W}}_{g}{\text{N}}_{gn}\right)}^{2013}+{\left({\text{W}}_{g}{\text{N}}_{gn}\right)}^{2014}$$

$${\text{WN}}_{1}={\left({\text{W}}_{g}{\text{N}}_{gn}\right)}^{2015}+{\left({\text{W}}_{g}{\text{N}}_{gn}\right)}^{2016}$$

$$\text{Code}\;\text{Count}\;\text{Effect}\;\text{(CCE)}={\left[\left({\text{WN}}_{1}-{\text{WN}}_{\text{0}}\right)*\left({\text{W}}_{g}-{\text{CMI}}_{ref}\right)\right]}^{2}$$

$$\text{Code}\;\text{attributable}\;\text{change}\;=\left(\frac{{\text{CCE}}_{g}}{\Sigma \text{CCE}}\right)*100\%$$

Where W _g_ is the weight calculated for each ICD or CPT, and N _gn_ is the number of cases within each ICD in the total population.

CMI *share* change formula:

$$\text{Code}\;\text{share}\;\text{change}\;=\frac{\left({\text{WN}}_{\text{1}}-{\text{WN}}_{\text{0}}\right)}{{\text{WN}}_{\text{0}}}$$

## Results

### Descriptive statistics

The total study population across 2011–2016 included 1,353,025 inpatient hospitalizations, or about 230,000 per year, which were composed of 55% medical cases, 43% surgical cases and 2% mixed cases (see Table 1). Medium-stay medical cases were about four times more frequent than short-stay cases (43% to 11%), with long-stay cases being a small minority (1%). Among surgical cases the ratio of case to procedure was 1 case to 1.1 procedure, and among mixed cases 1 case to 1.5 procedure.

There was limited variation across 2011–2016 in terms of total cases, the exception being 2011, during which not all hospitals were transmitting discharge diagnoses to the MoPH hospitalization database until June 2011. We observed a minor decrease of surgical cases (absolute and relative) and medical short-stay cases in 2015 and 2016.

Approximately one in every three hospitalizations occurs in public hospitals. The proportion of hospitalization at public hospitals increased from 31% to 37% between 2012 and 2016. Most of this change is due to increased medical cases (32% to 41%) and, to a lesser extent, increasing surgical cases in public hospitals (30% to 33%).

### Impact on CMI level and trend, across public and private hospitals (Objective 1)

*Pre-intervention*

The pre-intervention CMI monthly coefficients were 0.975, 1.284 and 1.783 for each of medical, surgical and mixed cases, respectively. Among medical cases, CMI was lowest for short-stays (0.352) and highest for long-stay cases (3.326).

*Post-intervention*

**Figure 1.**
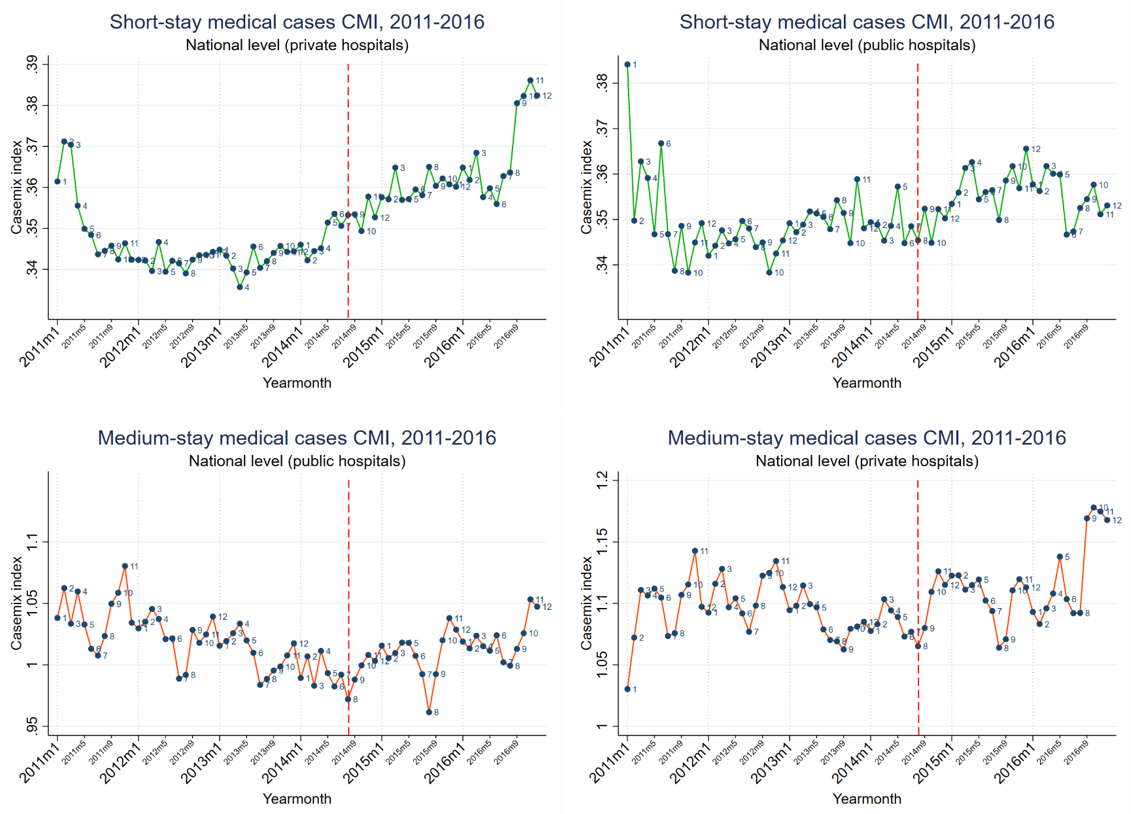
Medical short and medium-stay hospitalizations monthly casemix index (CMI), at public and private hospitals, unadjusted, 2011–2016.

**Figure 2.**
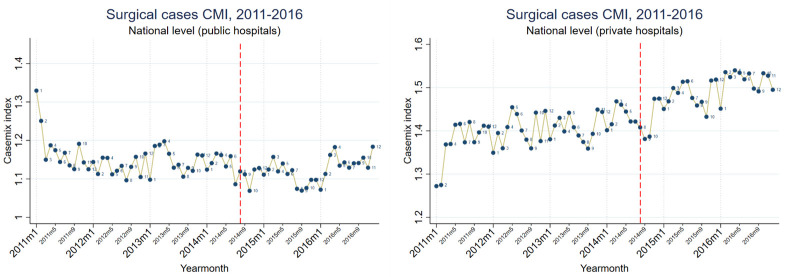
Surgical hospitalizations monthly casemix index (CMI), at public and private hospitals, 2011–2016.

Overall the intervention had variable impacts depending on case type and hospital ownership, resulting in either increased or unchanged CMI (see Table 2, Figure 1 and Figure 2).

**Table 2.** Results of the interrupted time-series analysis (ITSA) on casemix index at Lebanese hospitals, adjusted for seasonality, 2011–2016, with the intervention point of August 2014 (two-month effect lag).

a. Summary of changes									
Case type		Hospitals	Before intervention			After intervention			
			Monthly CMI coefficient	TREND		TREND		LEVEL	
				% (CI)	*Explained by*	% (CI)	*Explained by*	% (CI)	*Explained by*
**Medical**		All	0.975	➘0.10% (0.06 - 0.13%)	*Medium-stay* *cases*	➚0.11% (0.02 - 0.21%)	*Medium-stay cases*	⬆2.25% (0.51 - 3.98%)	*Short-stay cases*
		Public	0.941	➘0.17% (0.11 - 0.23%)		➚0.15% (0.06 - 0.22%)	-	-	-
		Private	0.989	➘0.06% (0.01 - 0.11%)		➚0.19% (0.06 - 0.32%)	*Short-stay cases*	⬆2.70% (0.15 - 5.24%)	*Short-stay cases*
**Surgical**		All	1.284	➚0.05% (0.01 - 0.10%)	*-*	➚0.14% (0.06 - 0.21%) ^1^	-	-	-
		Public	1.179	-	*No trend*	➚0.13% (0.02 - 0.24%)	-	-	-
		Private	1.326	➘0.12% (0.03 - 0.21%)	*-*	➚0.24% (0.13 - 0.35%) ^2^	-	-	-
**Mixed**		All	1.783	-	*No trend*	-	*No trend*	-	-
		Public	1.964	-		-	*No trend*	-	-
		Private	1.689	-		➚0.35% (0.10 - 0.60%) ^3^	-	-	-
^1^p=0.06, ^2^p=0.11, ^3^p=0.33; no significant change between pre and post-intervention									
b. Absolute changes in casemix index									
Hospitals	ITSA aspect	MEDICAL				SURGICAL	MIXED		
		*All stays*	*Short-stay*	*Medium-stay*	*Long-stay*		*All components*	*Medical component*	*Surgical* *component*
**All**	Starting level	0.975	0.352	1.078	3.326	1.284	1.783	1.530	1.991
	Level at two months post- intervention	0.022	0.009	-	0.417	-	-	-	-
	Monthly trend, pre-intervention	-0.001	-	-0.001	-	0.001	-	-	-
	Monthly trend, post-intervention	0.001	0.001	0.001	-	0.002	-	-	-
	**Monthly trend,** **change**	0.002	0.001	0.002	-	-	-	-	-
**Public**	Starting level	0.941	0.356	1.049	2.697	1.179	1.964	1.708	2.200
	Level at two months post- intervention	-	0.007	-	-	-	-	-	-
	Monthly trend, pre-intervention	-0.002	-	-0.001	-	-	-	-	-
	Monthly trend, post-intervention	0.001	-	0.001	-	0.002	-	-0.014	-
	**Monthly trend,** **change**	0.003	-	0.003	-	0.003	-	-	-
**Private**	Starting level	0.989	0.352	1.089	3.498	1.326	1.689	1.421	1.906
	Level at two months post- intervention	0.027	0.009	-	0.489	-	-	-	-
	Monthly trend, pre-intervention	-0.001	-	-	-	0.002	-	-	-
	Monthly trend, post-intervention	0.002	0.001	0.002	-	0.003	0.006	-	0.008
	**Monthly trend,** **change**	0.003	0.001	0.002	-	-	-	-	-
(Numerating only results significant at p<0.05)									
c. Relative changes, using respective monthly casemix index starting level, percentages									
Hospitals	ITSA aspect	MEDICAL				SURGICAL	MIXED		
		*All stays*	*Short-stay*	*Medium-stay*	*Long-stay*		*All components*	*Medical component*	*Surgical* *component*
**All**	Starting level	0.975	0.352	1.078	3.326	1.284	1.783	1.530	1.991
	Level at two months post- intervention	2.25%	2.61%	-	0.125	-	-	-	-
	Monthly trend, pre-intervention	-0.10%	-	-0.06%	-	0.05%	-	-	-
	Monthly trend, post-intervention	0.11%	0.14%	0.09%	-	0.14%	-	-	-
	**Monthly trend,** **change**	0.22%	0.17%	0.16%	-	-	-	-	-
**Public**	Starting level	0.941	0.356	1.049	2.697	1.179	1.964	1.708	2.200
	Level at two months post- intervention	-	2.05%	-	-	-	-	-	-
	Monthly trend, pre-intervention	-0.17%	-	-0.12%	-	-	-	-	-
	Monthly trend, post-intervention	0.15%	-	0.11%	-	0.13%	-	-0.81%	-
	**Monthly trend,** **change**	0.31%	-	0.24%	-	0.22%	-	-	-
**Private**	Starting level	0.989	0.352	1.089	3.498	1.326	1.689	1.421	1.906
	Level at two months post- intervention	2.70%	2.56%	-	13.98%	-	-	-	-
	Monthly trend, pre-intervention	-0.06%	-	-	-	0.12%	-	-	-
	Monthly trend, post-intervention	0.19%	0.23%	0.15%	-	0.24%	0.35%	-	0.41%
	**Monthly trend,** **change**	0.25%	0.28%	0.18%	-	-	-	-	-
d. Confidence intervals for table 2b.									
Hospitals	ITSA aspect	MEDICAL				SURGICAL	MIXED		
		*All stays*	*Short-stay*	*Medium-stay*	*Long-stay*		*All components*	*Medical component*	*Surgical* *component*
**All**	Starting level	0.9599 - 0.9896	0.3416 - 0.3630	1.0498 - 1.1068	2.5017 - 4.1500	1.2666 - 1.3013	1.6649 - 1.9012	1.3860 - 1.6741	1.8782 - 2.1043
	Level at two months post- intervention	0.0050 - 0.0388	0.0031 - 0.0153	-	0.1414 - 0.6932	-	-	-	-
	Monthly trend, pre-intervention	(0.0013 - 0.0006)	-	(0.0012 - 0.0003)	-	0.0001 - 0.0013	-	-	-
	Monthly trend, post-intervention	0.0002 - 0.0020	0.0002 - 0.0007	0.0002 - 0.0017	-	0.0008 - 0.0027	-	-	-
	**Monthly trend,** **change**	0.0012 - 0.0030	0.0002 - 0.0010	0.0008 - 0.0026	-	-	-	-	-
**Public**	Starting level	0.9095 - 0.9723	0.3394 - 0.3728	1.0377 - 1.0593	1.3111 - 4.0834	1.0898 - 1.2691	1.7092 - 2.2188	1.5207 - 1.8947	1.8338 - 2.5664
	Level at two months post- intervention	-	0.0012 - 0.0135	-	-	-	-	-	-
	Monthly trend, pre-intervention	(0.0022 - 0.0010)	-	(0.0017 - 0.0010)	-	-	-	-	-
	Monthly trend, post-intervention	0.0006 - 0.0021	-	0.0009 - 0.0015	-	0.0002 - 0.0028	-	(0.0254 - 0.0024)	-
	**Monthly trend,** **change**	0.0019 - 0.0039	-	0.0020 - 0.0030	-	0.0007 - 0.0046	-	-	-
**Private**	Starting level	0.9657 - 1.0117	0.3409 - 0.3628	1.0523 - 1.1258	2.7808 - 4.2154	1.2820 - 1.3702	1.6016 - 1.7767	1.2363 - 1.6055	1.8291 - 1.9833
	Level at two months post- intervention	0.0015 - 0.0518	0.0012 - 0.0169	-	0.1433 - 0.8347	-	-	-	-
	Monthly trend, pre-intervention	(0.0011 - 0.0001)	-	-	-	0.0004 - 0.0028	-	-	-
	Monthly trend, post-intervention	0.0006 - 0.0032	0.0004 - 0.0013	0.0002 - 0.0030	-	0.0017 - 0.0047	0.0017 - 0.0101	-	0.0018 - 0.0139
	**Monthly trend,** **change**	0.0012 - 0.0038	0.0004 - 0.0016	0.0004 - 0.0035	-	-	-	-	-
(Numerating only results significant at p<0.05)									

a. Medical cases

A decreasing pre-intervention trend was reversed, resulting in an increasing trend across all hospitals. Large level changes in CMI were also found at two months post-intervention, among public and private hospitals. No level changes were found among medium-stay cases. Overall, the main impact was on short and medium-stay cases, specifically a level change in the former and a trend change in the latter.

b. Surgical cases

An increasing pre-intervention trend continued unchanged in the post-intervention period across all hospitals. However, among public hospitals the absence of a pre-intervention trend was replaced with an increasing CMI trend.

c. Mixed cases

The only change found among mixed cases was an increasing trend in the medical component of private hospital cases. This had minimal impact on the overall CMI, as mixed cases comprised a very modest proportion of all hospitalizations.

We observed seasonality in medical CMI, and in particular medium-stay cases, with a March minor peak, a July–August trough, and a November–December major peak. Surgical casemix seasonality was limited to private hospitals only, with an April–June major peak, and a November–December minor peak. Mixed casemix seasonality had March and October peaks.

### Changes in diagnoses and procedures (Objective 2)

The number of medical case discharge ICD codes used across 2013–2016 was 3,164 for short-stay, 5,828 for medium-stay and 1,566 for long-stay cases, and 3,911 CPT codes for surgical cases. The 20 selected codes in each case type accounted for a majority of the change in CMI (see Table 3).

**Table 3.** Summary results of casemix index (CMI) changes due diagnoses and procedures within different case types.

Case type		Number of codes (n)	Top 20 codes proportion of total cases (%)	Range of change in CMI share per code (%)
**Medical**	**Short-stay**	3,164	94.1%	-3.1% to 0.6%
	**Medium-stay**	5,828	80.6%	-0.8% to 1.4%
	**Long-stay**	1,566	55.8%	-0.7% to 1.4%
**Surgical**		3,911	96.2%	-0.6% to 1.3%

### CMI change

Codes with the greatest changes from pre- to post-intervention are displayed in Table 4 (for detailed changes see Table 5). These included 2,970 fewer cases of abdominal and pelvic pain, 698 fewer cases of intestinal infectious diseases, 1,001 fewer cases of fever of unknown origin, and 783 fewer cases of essential hypertension; altogether this represents about 4.3% of all annual medical cases. The greatest change on medium-stay CMI was due to a decrease of 2,237 cases of diarrhea and gastroenteritis. Two forms of shifting were also noted among several codes: from medium to short-stay cases (e.g. A09, I10, I20-I25.9), and from three-digit to four-digit ICD10 codes (e.g. J18, J44, P22). The greatest change on short-stay CMI was due to the improved coding of chemotherapy cases under the relevant code (Z51.1) rather than under neoplasms (C00-D49).

**Table 4.** Diagnoses and procedures with the greatest change effect on casemix index (CMI), pre- versus post-intervention.

Description	ICD/CPT code	Major effects	Notes
**Neoplasms**	C00-D49	Increased ss-CMI (87%)	Greatest change on ss-CMI ⬇10,179 cases, net
			Mainly due to malignant neoplasm of breast and acute lymphoblastic leukemia
			Concurrent with increase in chemotherapy Z51.1 ⬆11,666 cases
**Intestinal infectious diseases** **(category)**	A00-A09		⬇961 ms-cases; ⬆263 ss-cases
**Diarrhea and gastroenteritis of** **presumed infectious origin**	A09	Increased ms-CMI (25%)	Greatest change on ms-CMI ⬇2,237 ms-cases; ⬆179 ss-cases
**Unspecified non-infective** **gastroenteritis and colitis**	K52.9	Decreased ms-CMI (2%)	⬇745 ms-cases; ⬇108 ss-cases
**Abdominal and pelvic pain (category)**	R10-R10.4		⬆2,970 ms-cases, net
**Abdominal and pelvic pain, other/** **unspecified abdominal pain**	R10, R10.4	Increased ms- and ss-CMI	⬇1,975 ms-cases; ⬆174 ss-cases
**Influenza and pneumonia**	J09-J18	Decreased ms-CMI (4%)	⬆3,909 ms-cases; ⬆298 ss-cases
**Pneumonia, non-specific**	J18		⬇1,456 ms-cases
**Pneumonia, specific**	J18.0, J18.9		⬆4,692 ms-cases
**COPD**	J44-J44.9	Increased ms-CMI (5%)	⬆1,306 ms-cases, net
**COPD with acute exacerbation**	J44.1		⬆625 ms-cases
**COPD, non-specific**	J44		⬇234 ms-cases
**Acute bronchitis**	J20-J20.9	Decreased ms-CMI (3%)	⬆1,145 ms-cases
	J20		⬆747 ms-cases
**Essential hypertension**	I10		⬇957 ms-cases; ⬆174 ss-cases
**Ischemic heart diseases**	I20-I25.9	Decreased ms-CMI (7%)	⬇1,100 ms-cases; ⬆275 ss-cases
			Mainly due to angina pectoris and acute myocardial infarction
**Fever of unknown origin**	R50	Increased ms-CMI (3%)	⬇989 ms-cases; ⬇12 ss-cases
**Stroke**	I64	Decreased ms-CMI (2%)	⬇383 ms-cases; ⬆12 ss-cases
**Respiratory distress of newborn,** **non-specific**	P22		⬇334 ms-cases
**Respiratory distress of newborn,** **specific**	P22.0		⬆287 ms-cases
**Vaginal delivery**	F9410L1	Increased surgical CMI (43%)	⬇3,939 cases
			Greatest change in absolute and in CMI share among all ICD/CPT codes
**Percutaneous Transluminal Coronary** **Angioplasty (PTCA)**	X2983/6	Increased surgical CMI (36%)	⬆778 cases

ss, short-stay; ms, medium-stay; COPD, chronic obstructive pulmonary disease; ICD, International Classification of Diseases; CPT, Common Procedural Terminology.

**Table 5.** Identifying diagnoses/procedures with the greatest change in terms of casemix index, from pre-intervention to post-intervention.

a. Short-stay medical cases								
#	ICD code	Cases (n) 2013–2014	Cases (n) 2015–2016	Change in cases (n)	Change in cases (%)	Change in CMI share, relative	Change in CMI, relative	Description
*1*	C50	5,776	3,384	-2,392	-41.4%	-3.1%	43.3%	Malignant neoplasm of breast
*2*	C91.0	2,873	1,223	-1,650	-57.4%	-2.4%	19.9%	Acute lymphoblastic leukaemia
*3*	C76	297	89	-208	-70.0%	-0.6%	5.9%	Malignant neoplasm of other and ill-defined sites
*4*	C50.9	5,550	4,744	-806	-14.5%	-0.5%	5.2%	Breast, unspecified
*5*	C91	680	130	-550	-80.9%	-0.7%	5.1%	Lymphoid leukaemia
*6*	E88.0	92	2	-90	-97.8%	-0.4%	4.0%	Disorders of plasma-protein metabolism, not elsewhere classified
*7*	C90.0	1,135	474	-661	-58.2%	-0.9%	3.7%	Multiple myeloma
*8*	C25.9	524	130	-394	-75.2%	-0.6%	1.9%	Pancreas, unspecified
*9*	Z51	427	21	-406	-95.1%	-0.6%	1.5%	Other medical care
*10*	I10	697	871	174	25.0%	0.6%	0.7%	Essential (primary) hypertension
*11*	C18.9	1,046	691	-355	-33.9%	-0.5%	0.4%	Colon, unspecified
*12*	Z51.8	6	106	100	1666.7%	0.1%	0.3%	Other specified medical care
*13*	I46.9	82	103	21	25.6%	0.2%	0.3%	Cardiac arrest, unspecified
*14*	C56	1,414	947	-467	-33.0%	-0.6%	0.3%	Malignant neoplasm of ovary
*15*	N18	86	134	48	55.8%	0.2%	0.3%	Chronic renal failure
*16*	I25.1	13	38	25	192.3%	0.1%	0.3%	Atherosclerotic heart disease
*17*	I21.9	25	49	24	96.0%	0.1%	0.3%	Acute myocardial infarction, unspecified
*18*	I20.0	177	244	67	37.9%	0.3%	0.3%	Unstable angina
*19*	A09	947	1,126	179	18.9%	0.5%	0.3%	Diarrhoea and gastroenteritis of presumed infectious origin
*20*	I48	77	142	65	84.4%	0.2%	0.3%	Atrial fibrillation and flutter
b. Medium-stay medical cases								
#	ICD code	Cases (n) 2013-2014	Cases (n) 2015-2016	Change in cases (n)	Change in cases (%)	Change in CMI share, relative	Change in CMI, relative	Description
*1*	A09	18,440	16,203	-2,237	-12.1%	-0.8%	25.0%	Diarrhoea and gastroenteritis of presumed infectious origin
*2*	I21	745	350	-395	-53.0%	-0.5%	7.8%	Acute myocardial infarction
*3*	P22.0	420	707	287	68.3%	0.3%	6.1%	Respiratory distress syndrome of newborn
*4*	J18.0	6,487	10,134	3,647	56.2%	1.4%	5.6%	Bronchopneumonia, unspecified
*5*	J44.1	439	1,064	625	142.4%	0.5%	5.0%	Chronic obstructive pulmonary disease with acute exacerbation, unspecified
*6*	R10	11,406	10,034	-1,372	-12.0%	-0.6%	4.4%	Abdominal and pelvic pain
*7*	I46.9	127	262	135	106.3%	0.2%	3.3%	Cardiac arrest, unspecified
*8*	R50	3,348	2,359	-989	-29.5%	-0.4%	2.8%	Fever of unknown origin
*9*	K52.9	2,343	3,088	745	31.8%	0.2%	2.4%	Noninfective gastroenteritis and colitis, unspecified
*10*	Z38.0	75	262	187	249.3%	0.2%	2.4%	Singleton, born in hospital
*11*	P22	1,369	1,035	-334	-24.4%	-0.3%	2.4%	Respiratory distress of newborn
*12*	J20	2,291	3,038	747	32.6%	0.2%	2.4%	Acute bronchitis
*13*	R07.0	306	137	-169	-55.2%	-0.2%	2.0%	Pain in throat
*14*	A41.9	525	708	183	34.9%	0.2%	1.8%	Septicaemia, unspecified
*15*	I64	1,454	1,071	-383	-26.3%	-0.3%	1.7%	Stroke, not specified as haemorrhage or infarction
*16*	A08.4	206	804	598	290.3%	0.2%	1.3%	Viral intestinal infection, unspecified
*17*	J18.9	2,079	3,124	1,045	50.3%	0.6%	1.2%	Pneumonia, unspecified
*18*	J98	166	3	-163	-98.2%	-0.2%	1.1%	Other respiratory disorders
*19*	R10.4	3,167	2,564	-603	-19.0%	-0.2%	1.1%	Other and unspecified abdominal pain
*20*	G81.9	428	198	-230	-53.7%	-0.2%	1.0%	Hemiplegia, unspecified
c. Long-stay medical cases								
#	ICD code	Cases (n) 2013-2014	Cases (n) 2015-2016	Change in cases (n)	Change in cases (%)	Change in CMI share, relative	Change in CMI, relative	Description
*1*	P22	215	108	-107	-49.8%	-0.4%	17.9%	Respiratory distress of newborn
*2*	F20.9	1	31	30	3000.0%	1.4%	4.9%	Schizophrenia, unspecified
*3*	F25.0	2	26	24	1200.0%	1.1%	3.1%	Schizoaffective disorder, manic type
*4*	A41.9	74	121	47	63.5%	0.3%	2.7%	Septicaemia, unspecified
*5*	I20	74	32	-42	-56.8%	-0.2%	2.5%	Angina pectoris
*6*	F10	39	6	-33	-84.6%	-0.1%	2.5%	Mental and behavioural disorders due to use of alcohol
*7*	F11	45	13	-32	-71.1%	0.0%	2.4%	Mental and behavioural disorders due to use of opioids
*8*	G81	49	11	-38	-77.6%	-0.2%	2.4%	Hemiplegia
*9*	J98	42	0	-42	-100.0%	-0.2%	2.2%	Other respiratory disorders
*10*	I50	139	102	-37	-26.6%	-0.1%	2.1%	Heart failure
*11*	F20.0	88	58	-30	-34.1%	0.0%	2.0%	Paranoid schizophrenia
*12*	Z38.0	18	55	37	205.6%	0.2%	1.7%	Singleton, born in hospital
*13*	P22.0	114	149	35	30.7%	0.3%	1.5%	Respiratory distress syndrome of newborn
*14*	F11.2	0	16	16		0.7%	1.4%	Mental and behavioural disorders due to use of opioids, dependence syndrome
*15*	M96.9	16	0	-16	-100.0%	-0.7%	1.4%	Postprocedural musculoskeletal disorder, unspecified
*16*	F25	45	21	-24	-53.3%	0.0%	1.3%	Schizoaffective disorders
*17*	R10.0	28	6	-22	-78.6%	0.0%	1.0%	Acute abdomen
*18*	I46.9	38	73	35	92.1%	0.3%	1.0%	Cardiac arrest, unspecified
*19*	J18.9	72	99	27	37.5%	0.2%	0.9%	Pneumonia, unspecified
*20*	G45.9	13	37	24	184.6%	0.1%	0.9%	Transient cerebral ischaemic attack, unspecified
d. Surgical cases								
#	CPT code	Cases (n) 2013-2014	Cases (n) 2015-2016	Change in cases (n)	Change in cases (%)	Change in CMI share, relative	Change in CMI, relative	Description
*1*	F9410L1	18,833	14,894	-3,939	-20.9%	-0.6%	43.4%	Vaginal delivery only including postpartum care
*2*	X2986L1	1,153	1,687	534	46.3%	1.3%	33.7%	Percutaneous transluminal coronary angioplasty: with stent; each additional vessel
*3*	M7447G1	745	954	209	28.1%	0.5%	4.9%	Arthroplasty, knee condyle and plateau ("total knee replacement")
*4*	C3510G	1,323	1,179	-144	-10.9%	-0.3%	3.2%	Coronary artery bypass, vein only: any number W/CPB
*5*	X2983L1	3,154	3,398	244	7.7%	0.5%	2.2%	Percutaneous transluminal coronary angioplasty: with stent
*6*	C3000Gb	423	809	386	91.3%	0.4%	1.5%	Cardiopulmonary bypass, including cannulation: add to primary procedure (W/CPB)
*7*	X3548L	11,900	12,556	656	5.5%	0.2%	1.1%	Combined left heart catheterization, selective coronary angiography, one or more.
*8*	C3411G	189	115	-74	-39.2%	-0.2%	1.0%	Replacement, aortic valve: w/wout aortic annulus enlargement, mechanical.
*9*	M7125G1	294	447	153	52.0%	0.2%	0.7%	Partial hip replacement, prosthesis (eg, femoral stem prosthesis, bipolar arthroplasty)
*10*	R0140Gb	3,600	3,959	359	10.0%	0.0%	0.7%	Submucous resection turbinate, complete or partial
*11*	M2842G1	346	450	104	30.1%	0.2%	0.7%	Spinal instrumentation, posterior: segmental fixation.
*12*	M7130G1	622	715	93	15.0%	0.2%	0.6%	Arthroplasty, acetabular and proximal femoral prosthetic replacement (Age <60y)
*13*	F9812G	3,386	2,959	-427	-12.6%	-0.1%	0.5%	Treatment of incomplete or missed abortion, any trimester, completed surgically
*14*	M2843G1	108	182	74	68.5%	0.1%	0.4%	Spinal instrumentation, posterior: segmental fixation
*15*	X2987L	23	58	35	152.2%	0.1%	0.4%	Closure of PDA or ASD by Amplatzer
*16*	C3411Ga	44	9	-35	-79.5%	-0.1%	0.3%	Replacement, aortic valve: w/wout aortic annulus enlargement, biological.
*17*	C5301G	132	94	-38	-28.8%	-0.1%	0.2%	Thromboendarterectomy, with or without patch graft
*18*	U2332Gb	656	852	196	29.9%	0.0%	0.2%	Cystourethroscopy, with insertion of indwelling ureteral stent
*19*	D2820G	2,651	2,349	-302	-11.4%	0.0%	0.2%	Tonsillectomy with or without adenoidectomy
*20*	M7715G	111	25	-86	-77.5%	-0.1%	0.2%	Osteoplasty, tibia and fibula, lengthening

ICD, International Classification of Diseases; CPT, Common Procedural Terminology; CMI, casemix index; CPB, cardiopulmonary bypass; PDA, patent ductus arteriosus; ASD, atrial septal defect.

Given the magnitude of the change in vaginal deliveries, we further examined deliveries throughout 2013–2016 (vaginal and cesarean section). Private hospitals had 36.9% less vaginal deliveries (4,022) in 2015–2016 than in 2013–2014, while public hospitals increased by less than 1%. The decreasing trend in private hospitals began in early 2014, which was prior to the P4P intervention in late 2014. Concurrently, cesarean deliveries decreased at private hospitals by 7.5% and increased at public hospitals by 9.2%. The latter change also had an impact in increasing surgical CMI at public hospitals. Overall, vaginal deliveries under MoPH coverage decreased by 21.0% (18,843 to 14,894), while cesarean deliveries decreased by 2.5% in the aforementioned two-year periods (23,607 to 23,011).

### CMI share change

The range of change of CMI share per code from pre- to post-intervention is shown in Table 3. The conditions with the greatest change in their share of CMI included: percutaneous transluminal coronary angioplasty (PTCA) and vaginal delivery (surgical); malignant neoplasm of breast and acute lymphoblastic leukemia (short-stay); bronchopneumonia, diarrhea and gastroenteritis (medium-stay) (see Table 5).

## Discussion

This study investigated the impact of integrating a P4P policy on the complexity of hospitalizations at hospitals contracted by the Lebanese Ministry of Public Health. We found that the intervention resulted in increased case complexity, specifically among medical cases. We used a systematic approach, first examining all hospitalizations by case type, lengths of stay and hospital ownerships for changes, and subsequently identifying and quantifying which diagnoses and procedures explain this change.

Case complexity was greatest for mixed cases, followed by surgical and medical cases. This was unsurprising considering that mixed cases are typically more complex and include ICU cases. Furthermore, a large proportion of medical cases are for short-term evaluation, basic treatment, or chemotherapy. Similar reasoning explains medical casemix increasing with longer stays.

### Medical casemix: decreased unnecessary hospitalizations and improved coding

During the pre-intervention period, medical casemix had a decreasing trend, possibly as a result of increasing unnecessary hospitalizations, for which an incentive exists. The intervention resulted in a reversal to an increasing casemix trend, as well as a short-term level change, at both public and private hospitals. The trend and level changes are attributable to medium and short-stay cases, respectively.

The decrease in diarrhea and gastroenteritis cases had a greater effect on medium-stay casemix than any other condition. Diarrhea and gastroenteritis cases are likely to be responsible for more unnecessary hospitalizations than any other diagnosis ( Kronfol *et al*., 2014; To *et al*., 1996). Similar decreases were also found in abdominal and pelvic pain, essential hypertension cases and fever of unknown origin, all being major sources of unnecessary hospitalizations ( Kronfol *et al*., 2014). It is relevant to highlight that some of these conditions are more precisely symptoms (e.g. pain), which are not generally appropriate as discharge diagnoses and likely to be unnecessary hospitalizations. We find that the intervention decreased unnecessary hospitalizations by changing admission practices for these four conditions. The change in absolute cases for these conditions were at least one order of magnitude greater than any such changes since 2011 and are unlikely to be due to decreased disease burden.

We also found large shifts from medium to short-stay cases among each of diarrhea and gastroenteritis, abdominal and pelvic pain and essential hypertension cases. This suggests that hospitals not only decreased unnecessary hospitalizations, but also decreased unnecessary hospital stays. Although changes in length of stay are often difficult to associate with quality of care, among such conditions it is likely a result of improved hospital practices during hospitalization, in addition to the aforementioned improved pre-hospitalization practices. Such changes were seen among both public and private hospitals.

The large increase in chronic obstructive pulmonary disease (COPD) cases may be influenced by both practice changes and increasing burden. A steady increase in global COPD has been noted, with smoking prevalence and air pollution being the major risk factors ( Lopez *et al*., 2006). These two factors are also prevalent in Lebanon, and are likely to have contributed to the increased COPD burden ( IHME, 2018). It is also possible that improved hospital admission practices influenced this increase. The increases in pneumonia and acute bronchitis cases are likely due to increased disease burden, with yearly and seasonal variations typical of infectious disease. We note the possibility of COPD acute exacerbation of COPD being misdiagnosed as acute bronchitis, as well as acute bronchitis also having smoking as a risk factor.

Given that this intervention had no direct impact on the separately pre-determined hospital budgets, it may be argued that the decreases in unnecessary hospitalizations provided hospitals with greater resources to hospitalize more necessary hospitalizations, such as COPD, pneumonia and acute bronchitis. Confirmation of such an impact would, however, require further investigation.

The decrease in ischemic heart disease cases, including a length of stay shift, may be associated with the concurrent increase in percutaneous transluminal coronary angioplasty (PTCA), which is indicated for certain ischemic heart disease cases. PTCA over-utilization has been documented in other healthcare systems and has had decreasing use in recent years ( Mariotto *et al*., 1999; Weiss & Elixhauser, 2014). It is not possible to evaluate medically indicated PTCA cases in this study, and further investigation would be required to ascertain the level of benefit or abuse.

The code-level findings indicate improved coding practices, which has been documented elsewhere to occur alongside real case changes following the introduction of CMI ( Ginsburg & Carter, 1986; Goldfarb & Coffey, 1992). The improved coding for chemotherapy, particularly in breast cancer and leukemia, explained most of the short-stay casemix increase. Among medium and short-stays we find a shift to full coding digits and more specific codes, specifically among neoplasms, pneumonia, COPD, respiratory distress of newborn, and diarrhea and gastroenteritis.

### Surgical casemix

Changes in surgical casemix were limited to public hospitals only, with an increasing trend emerging in the post-intervention period. This was explained by increased cesarean deliveries in public hospitals, which compensated a concurrent decrease in private hospitals, and was identified as a trend preceding the intervention by several months, and therefore not likely impacted by the intervention itself.

Private hospitals continued their increasing casemix trend without any change following the intervention. This trend was explained by two factors: increasing PTCA cases throughout 2011–2016 and decreasing vaginal deliveries since early 2014. The former may also be an example of private hospitals being faster adopters of new technologies than public hospitals or having greater potential for over-utilization. We had expected to find new (and costlier) procedures such as PTCA to explain a large proportion of an increasing surgical casemix at all hospitals. However, with the exception of PTCA, adoption of new procedures had a negligible effect on surgical casemix.

The large decrease in vaginal deliveries at private hospitals coincides with the sharp increase into Lebanon of refugees from Syria in early 2014. This change was not compensated by public hospitals, whose vaginal deliveries remained generally unchanged. Deliveries had been documented in 2013 as the leading cause for hospitalization among Syrian refugees in Lebanon ( Huster *et al*., 2014). This suggests that, under MoPH coverage, Lebanese women having vaginal deliveries had decreased access to private hospitals, or otherwise opted to deliver outside of this coverage (out-of-pocket payment). The hospitalization access of refugee and citizen communities requires further investigation, including the role of private hospitals.

### Improvement potential, hospital ownership, and seasonality

Hospitals had a greater improvement space in medical cases than in surgical cases. Prior to the intervention, medical ICD10 coding had no link to reimbursement (fee-for-service), unlike surgical codes (flat-fee). The intervention incentivized improved medical coding (for accurate CMI assessment), but the same was not relevant for surgical coding.

Private hospitals had a greater overall casemix than public hospitals, suggesting that more complex cases were admitted to private hospitals, as had been observed in previous MoPH investigations ( Ammar *et al*., 2013). However, private and public hospitals had similar short-stay casemix, while public hospitals had a higher casemix for mixed cases. Two factors at private hospitals that are relevant to note are their possibly greater capacity for accurate coding and for healthcare technology. The former has been found elsewhere to explain some of the casemix gap between public and private hospitals, while the latter is associated with increased casemix ( Mendez *et al*., 2014; Park *et al*., 2017).

We also note the increasing proportion of hospitalizations at public hospitals throughout 2011–2016, which continues the trend observed by the MoPH for preceding years. Since the proportion of contracted public and private hospitals had remained relatively unchanged, this is attributable to the MoPH’s broad policy of increasing the service delivery carried out at public rather than private hospitals.

The seasonality in the casemix within different case types is likely explained by a combination of disease burden variation throughout the year, and pre-planned hospitalizations that avoid vacation periods. The November-December peak for medical medium-stay may be related to influenza seasonality in Lebanon ( World Health Organization, 2019). Further investigations would be required to associate casemix seasonality with specific diseases or conditions. It is noteworthy that surgical casemix seasonality was found only in private hospitals, peaking during the three months preceding the summer period, which suggests that pre-planned hospitalizations are more common in private than in public hospitals.

### P4P design

The importance of detailing P4P designs has been highlighted, particularly considering the heterogeneity of such interventions ( Chee *et al*., 2016). Notable design features of the MoPH P4P is the linkage between performance and reimbursement tier, rather than a bonus/penalty. To our knowledge this has not been undertaken elsewhere. In Lebanon, such a feature was a result of the political and financial non-feasibility of having other financial mechanisms to reward hospitals for improved performance. However, this has the benefit of being more sustainable in avoiding bonus financing and maintaining a system aspect (tiers) familiar to hospitals. In effect, the design integrated P4P into the system of determining hospital reimbursement tier ( Soucat *et al*., 2017).

Another feature is the inclusion of CMI directly within the performance scoring, rather than as a risk adjustor for other outcomes (e.g. readmissions). This focus on casemix was intended to address unnecessary hospitalization, as well as the absence of risk-adjustment in hospital assessment. It also formed an objective that may be influenced by a wide range of hospitalizations, rather than a narrow set.

The type and magnitude of incentives are also important factors in determining P4P impact. Having casemix incentivized outside of a prospective payment system or similar approach likely limited the potential of providers to engage in up-coding or otherwise game the system, as has been observed in other casemix evaluations ( Radu *et al*., 2010; Sukul *et al*., 2019). Such behavior is further limited as providers do not have access to the weights used in casemix evaluation.

### Strengths and limitations

The use of ITS analysis with a large number of pre and post-intervention data points allowed us to account for background effects in what is a complex environment. Such effects may include other interventions or events that may have impacted hospitalization practice or burden of disease. No relevant system interventions were carried out by the MoPH throughout 2011–2016, besides the new intervention evaluated in this work. Other quality-focused activities undertaken by hospitals individually have not been assessed, though these are less likely to have system-level impact.

We relied on the data that is collected and input at hospital-level, and subsequently used to calculate CMI. As such, the validity of the casemix results relies on the quality of data input, specifically ICD10 and CPT codes. The intervention of August 2014 included advice to hospitals regarding improved coding accuracy, whose short-term results have been detected with CMI level changes. However, we do not account for coding quality initiatives that may have occurred within hospitals.

We recognize that the inclusion of age and comorbidities in the calculation of casemix index would allow a more accurate measure of hospitalization case complexity. Within the Lebanese healthcare setting there currently is insufficient information for selecting diagnoses and procedures for adjustment by age. The absence of comorbidity is due to the lack of routine recording of this variable across most hospitals, and policymaker concerns regarding potential miss-use. Age and comorbidities remain important areas for future development of casemix calculation and pay-for-performance, but their absence does not negate the findings of the current investigation.

## Conclusions

This research suggests that the integration of a hospital performance-payment policy in 2014 increased the effectiveness of the healthcare system, primarily due to decreased unnecessary hospitalizations, as well as a decreased length of stay of such cases. An improvement in quality of care may subsequently benefit patient health and resource use (efficiency). The new policy also led to improved discharge coding quality. Although unrelated to effectiveness, the latter finding increases the validity of evidence and policymaking that makes use of such information, including but not limited to the P4P design. Changes in unnecessary hospitalizations took place at a gradual pace compared to the more immediate coding practice changes. We also confirm that CMI can be appropriate tool to detect changes in hospitalizations or performance improvement.

By using a systems perspective, we were able to investigate the impact across different hospitalization case types, length of stay and hospital ownership, and went further to quantify and attribute changes to specific diagnoses and procedures. We also identified issues relevant for further investigation and policymaking (e.g. vaginal deliveries, COPD, ischemic heart disease treatment).

A similar analytical approach using interrupted time series may be used in the evaluation of other interventions on the hospitalization system. The algorithms developed for this research may also be adapted to investigate specific issues, as well as by a regulator or payer to actively monitor hospitalization trends across code and hospital attributes. This would support keeping providers accountable and increase the responsiveness capacity to address non-desirable or harmful hospitalization practices, such as unnecessary hospitalizations.

Despite the generally unfavorable evidence regarding the effectiveness of P4P in hospital settings, our findings suggest that certain P4P designs may be effective in specific contexts, such as that of the MoPH and Lebanese hospitals. This may be in part due to its integration within the system, rather than as a stand-alone external intervention, in addition to the existing space for improvement. 

Future investigations would be required to evaluate longer-term impact, which would inform whether the impact was a one-time result of introducing a new policy, or if it retains sustainable benefits. This would necessarily require continued commitment and capacity-building by the MoPH towards this process.

Our findings suggest that effective hospital regulation can be achieved through the systematic collection and analysis of readily available routine data. Our analytical approach to such data reveals relevant patterns of change to performance measures. LMICs that lack casemix adjustment and incentives for improving hospital performance may choose to adopt similar approaches and monitoring systems to measure and improve hospital performance over time.

## Data availability

### Underlying data

The source data are owned by the Lebanese Ministry of Public Health and in line with the IRB approval granted for this study, the authors are not permitted to share the source patient-level data. In compliance with the MoPH’s obligation on data privacy, the underlying data are accessible in a de-identified form upon request to the Ministry of Public Health ( directorgeneral@moph.gov.lb), including a justification for the request.
