# Supplementary material for: Hospital performance and payment: impact of integrating pay-for-performance on healthcare effectiveness in Lebanon
Source: Wellcome Open Res. 2020 Dec 10;5:95. Originally published 2020 May 14. [Version 2] doi: 10.12688/wellcomeopenres.15810.2 (PMC7780336; doi:10.12688/wellcomeopenres.15810.2)
Supplement: Peer review history

# Peer review: Reviewer response for version 2

**DOI:** 10.21956/wellcomeopenres.18000.r41802

**Published:** 2020-12-18

**Authors:** Linghan Shan

**Affiliations:** 1 Department of Social Medicine, School of Public Health, Harbin Medical University, Harbin, Heilongjiang, China

Dear author, I think this is a high-quality study that can provide evidence for many policy researchers and policy makers.

**Is the work clearly and accurately presented and does it cite the current literature?**

 Yes

**Is the study design appropriate and is the work technically sound?**

 Yes

**Are sufficient details of methods and analysis provided to allow replication by others?**

 Yes

**If applicable, is the statistical analysis and its interpretation appropriate?**

 I cannot comment. A qualified statistician is required.

**Are all the source data underlying the results available to ensure full reproducibility?**

 No source data required

**Are the conclusions drawn adequately supported by the results?**

 Yes

Is the work clearly and accurately presented and does it cite the current literature?

Yes

If applicable, is the statistical analysis and its interpretation appropriate?

I cannot comment. A qualified statistician is required.

Are all the source data underlying the results available to ensure full reproducibility?

No source data required

Is the study design appropriate and is the work technically sound?

Yes

Are the conclusions drawn adequately supported by the results?

Yes

Are sufficient details of methods and analysis provided to allow replication by others?

Yes

Reviewer Expertise:

Health policy assessment; health insurance; health system reform

I confirm that I have read this submission and believe that I have an appropriate level of expertise to confirm that it is of an acceptable scientific standard.

# Peer review: Reviewer response for version 1

**DOI:** 10.21956/wellcomeopenres.17338.r39557

**Published:** 2020-08-10

**Authors:** Linghan Shan

**Affiliations:** 1 Department of Social Medicine, School of Public Health, Harbin Medical University, Harbin, Heilongjiang, China

Thank you for writing such a valuable and enlightening article, it is of an acceptable scientific standard, however, I have a number of small changes to the article, as follows:

1. In methods it was said that “We applied a systematic approach to assess the impact of the new policy on hospital performance.” What is the framework of the systematic approach, what are the differences from previous studies, or what is the basis for it? I see that your explanation of "systematic approach" is scattered in the article. I hope it can be more clearly expressed.
2. In the Introduction it was said “The MoPH is the largest public payer, covering hospitalization for about 52% of Lebanese, who otherwise lack any insurance coverage for hospitalization (Ammar, 2009).” What is the coverage now? I want to know this information because it is directly related to the impact of payment reform on hospitals. High coverage means that the payer has strong bargaining power, so the reform of payment mode will bring greater binding force to hospitals, otherwise, the binding force will be small.
3. I would like to see more details about the pre-reform payment mechanism, restrictive conditions of reform (such as you said “lack of local Diagnosis Related Groups (DRGs) on which most casemix systems rely on”), characteristics, advantages and disadvantages of the reform.
4. I see that your suggestions are all about recommending this reform measure. Do you have any relevant suggestions for the sustainable development of this reform measure? Is there a need for more supporting policies to further improve efficiency? Does this policy reform need further improvement?

Is the work clearly and accurately presented and does it cite the current literature?

Yes

If applicable, is the statistical analysis and its interpretation appropriate?

I cannot comment. A qualified statistician is required.

Are all the source data underlying the results available to ensure full reproducibility?

No source data required

Is the study design appropriate and is the work technically sound?

Yes

Are the conclusions drawn adequately supported by the results?

Yes

Are sufficient details of methods and analysis provided to allow replication by others?

Yes

Reviewer Expertise:

Health policy assessment; health insurance; health system reform

I confirm that I have read this submission and believe that I have an appropriate level of expertise to confirm that it is of an acceptable scientific standard, however I have significant reservations, as outlined above.

# Response

**Published:** 2020-10-06

**Authors:** Jade Khalife

**Affiliations:** Lund University, Sweden

*We are grateful for the valuable feedback received from the reviewer, which we have used to improve the manuscript. Kindly find below our responses on a point-by-point basis.*

- In methods it was said that “We applied a systematic approach to assess the impact of the new policy on hospital performance.” What is the framework of the systematic approach, what are the differences from previous studies, or what is the basis for it? I see that your explanation of "systematic approach" is scattered in the article. I hope it can be more clearly expressed.

*We have clarified our approach at the beginning of the discussion section to define what we mean by systematic approach. Our approach involved at a first stage examining all hospitalizations by case type, lengths of stay and hospital ownerships for casemix index changes. This was followed by a second stage for identifying and quantifying which diagnoses and procedures explain changes identified during the first stage.*

 

- In the Introduction it was said “The MoPH is the largest public payer, covering hospitalization for about 52% of Lebanese, who otherwise lack any insurance coverage for hospitalization (Ammar, 2009).” What is the coverage now? I want to know this information because it is directly related to the impact of payment reform on hospitals. High coverage means that the payer has strong bargaining power, so the reform of payment mode will bring greater binding force to hospitals, otherwise, the binding force will be small.

*The coverage in 2014 is expected to be similar (52%), according to the Ministry of Public Health. A more updated estimate is not available. However, this figure is expected to have increased since about 2018, as a consequence of increasing unemployment, which results in more citizens having coverage transferred from the National Social Security Fund to the Ministry of Public Health.*

 

- I would like to see more details about the pre-reform payment mechanism, restrictive conditions of reform (such as you said “lack of local Diagnosis Related Groups (DRGs) on which most casemix systems rely on”), characteristics, advantages and disadvantages of the reform.

*We have added a paragraph under ‘Policy reform’ to provide more detailed background information on the pre-reform period. The reform was used to categorize hospitals into the three reimbursement categories, however the medical cases remained reimbursed through fee-for-service, and surgical cases through a pre-defined flat-rate. The initiative had to solely utilize the reimbursement-performance link, but not hospital budgets, due to historical and political constraints. In the interest of space, we refer the reader to the following article where we have previously elaborated on some aspects: https://www.tandfonline.com/doi/full/10.1080/23288604.2016.1272979; ref.25, Khalife et al. 2017.*

 

- I see that your suggestions are all about recommending this reform measure. Do you have any relevant suggestions for the sustainable development of this reform measure? Is there a need for more supporting policies to further improve efficiency? Does this policy reform need further improvement?

*We have added some text under the conclusion towards this. The commitment of the MoPH to maintain the reimbursement-performance linkage is necessary, including ongoing evaluations and development. This would likely involve annual or bi-annual generation of hospital performance results, based on performance measures that have been proven beneficial and meaningful for inclusion.*

# Peer review: Reviewer response for version 1

**DOI:** 10.21956/wellcomeopenres.17338.r38761

**Published:** 2020-06-08

**Authors:** Kristiina Kahur

**Affiliations:** 1 Nordic Casemix Centre, Helsinki, Finland

Thank you for this very interesting research which is somehow unique on that field. Below are some comments and reflections which raised while reading the article:

- In conclusions it was said that “This increase was mainly attributed to decreased unnecessary hospitalizations and was accompanied by improved medical discharge coding practices”. I am wondering how was the improvement of coding assessed and is improved coding necessarily referring to increase of effectiveness?
- It was said in the article that the reimbursement was linked to a composite hospital total performance score (TPS) which means that the higher the CMI the higher the TPS. CMI is explicitly related to coding quality. It is known from different researches that coding activity is often incentivized by payments, thus this can incentivize the hospitals to increase the coding activity and perhaps up-coding in some cases which probably happened also in Lebanon. How much this could have been a case?
- Related to previous comment - in the article it was mentioned that “providers do not have access to the weights used in casemix evaluation”. This might limit the gaming and up-coding indeed. But on other hand it also makes the system non-transparent and weights used for calculation the CMI will be as a black box within the system which might have negative impact on improvement of coding quality.
- Given that there is no DRG system in place in Lebanon it somehow limited the development of CMI by using DRG cost-weights which would have been a conventional way when DRG system would have been used. The chosen approach was compensating the missing DRG cost-weights. However, it did not use the information/variables which would potentially have had impact on weights and CMI calculation. E.g. variable like age was not available for this study (as indicated in Data sources chapter) which would have had impact on weights in some cases. Also information about co-morbidities and complications was not taken into account which might be cost driver and thus, may have had impact on weights and CMI. It was also interesting that the medical cases where divided based on LOS whereas the surgical ones were not. In conventional cost-weight calculation also the cost/LOS outliers are taken into account which was not systematically the case in this study. Having DRG system in place, the calculations of CMI would be perhaps more consistent and internationally comparable by taking into account all possible variables a casemix system is using and which were not taken into account in this study.
- Given that five-year cost averages was used for calculation of CMI, how (if at all) was the inflation and other factors influencing the cost throughout the years taken into account? In other words, was the weight of one particular ICD or procedure code the same throughout the five-year period?
- It was mentioned in the article that the weight was calculated for each ICD (and procedure code). In table 4 it remains unclear if the separate weights were calculated e.g. for diagnosis code J44.1 and J44. Or was the separate weight calculated for codes J20-J20.9 and J20. However, table 5 which refers to diagnosis codes with the greatest change in terms of CMI, shows different values e.g. for code P22 and P22.0. This is somehow confusing because in general, subcodes of three-character diagnosis code should be clinically and economically meaningful and should have one weight, i.e. codes P22 and P22.0 should be similar in terms of clinical severity and cost. In general (and this really depends on national coding guidelines), if a three-character diagnosis code has subcodes, then the subcode as more specific one should be used instead of three-character code.

Is the work clearly and accurately presented and does it cite the current literature?

Yes

If applicable, is the statistical analysis and its interpretation appropriate?

I cannot comment. A qualified statistician is required.

Are all the source data underlying the results available to ensure full reproducibility?

No source data required

Is the study design appropriate and is the work technically sound?

Yes

Are the conclusions drawn adequately supported by the results?

Yes

Are sufficient details of methods and analysis provided to allow replication by others?

Yes

Reviewer Expertise:

Casemix systems' implementation, maintenance and development. Primary classifications. Coding quality. Contracting health care providers. Payment methods. Health financing reforms.

I confirm that I have read this submission and believe that I have an appropriate level of expertise to confirm that it is of an acceptable scientific standard.

# Response

**Published:** 2020-10-06

**Authors:** Jade Khalife

**Affiliations:** Lund University, Sweden

*We are grateful for the valuable feedback received from the reviewer, which we have used to improve the manuscript. Kindly find below our responses on a point-by-point basis.*

- In conclusions it was said that “This increase was mainly attributed to decreased unnecessary hospitalizations and was accompanied by improved medical discharge coding practices”. I am wondering how was the improvement of coding assessed and is improved coding necessarily referring to increase of effectiveness?

*Improved coding was assessed based on the changes in the coding of chemotherapy cases under the chemotherapy code (Z51.1), particularly breast cancer and leukemia, and the accompanied changes in the coding of neoplasms (C00-D49); this explained the greatest change on short-stay CMI. That these were indeed chemotherapy sessions, rather than hospitalization for investigation, was confirmed by their short length of stay (same/one-day) and their hospitalization cost. Effectively, this meant that hospitals were correctly coding chemotherapy sessions. Furthermore, we also found a shift to full coding digits and more specific codes, among short and medium-stay cases, specifically among neoplasms, pneumonia, COPD, respiratory distress of newborn, and diarrhea and gastroenteritis.*

*Our findings regarding improved coding were used to explain increased casemix index among short-stay cases, specifically the level changes. These do not refer to increased effectiveness, but would reflect improved code validity for use in the system. Improved effectiveness in our investigation refers to decreased unnecessary hospitalizations. We have clarified this in the ‘conclusions’ section.*

 

- It was said in the article that the reimbursement was linked to a composite hospital total performance score (TPS) which means that the higher the CMI the higher the TPS. CMI is explicitly related to coding quality. It is known from different researches that coding activity is often incentivized by payments, thus this can incentivize the hospitals to increase the coding activity and perhaps up-coding in some cases which probably happened also in Lebanon. How much this could have been a case?

*Although up-coding is generally a recognized issue in the Lebanese healthcare setting. However, given that providers were unaware of the TPS prior to the intervention in August 2014, we would not expect that there would have been an increase in up-coding above what are background levels. This similarly applies to increased coding activity, but only within surgical and mixed cases, where multiple-coding is used. Nonetheless, we acknowledge this factor will likely have to be more closely considered in future evaluations, with providers having become more aware of the incentive potential of up-coding, and other potential gaps to game the system.*

 

- Related to previous comment - in the article it was mentioned that “providers do not have access to the weights used in casemix evaluation”. This might limit the gaming and up-coding indeed. But on other hand it also makes the system non-transparent and weights used for calculation the CMI will be as a black box within the system which might have negative impact on improvement of coding quality.

*The lack of access to the weights used does limit transparency. However, based on past experience and under the existing conditions, this was considered by the Ministry of Public Health as an important feature to limit the abuse/miss-use of hospitalization among some providers. This has been possible to maintain thus far due to the joint commitment of the Syndicate of Private Hospitals towards the P4P initiative. Nevertheless, increasing transparency regarding weights and the TPS process would be important goals for future system development.*

 

- Given that there is no DRG system in place in Lebanon it somehow limited the development of CMI by using DRG cost-weights which would have been a conventional way when DRG system would have been used. The chosen approach was compensating the missing DRG cost-weights. However, it did not use the information/variables which would potentially have had impact on weights and CMI calculation. E.g. variable like age was not available for this study (as indicated in Data sources chapter) which would have had impact on weights in some cases. Also information about co-morbidities and complications was not taken into account which might be cost driver and thus, may have had impact on weights and CMI. It was also interesting that the medical cases where divided based on LOS whereas the surgical ones were not. In conventional cost-weight calculation also the cost/LOS outliers are taken into account which was not systematically the case in this study. Having DRG system in place, the calculations of CMI would be perhaps more consistent and internationally comparable by taking into account all possible variables a casemix system is using and which were not taken into account in this study.

*This is largely correct, although it is also pertinent to recognize the existing institutional context within which the CMI-based reimbursement system is developing. We have added a paragraph under ‘strengths and limitations’ to address age and comorbidities. We acknowledge that inclusion of age as a variable for casemix index calculation would be more advantageous. Its inclusion has been discussed at length with and within the MoPH. The conclusions of these discussions were that, although some diagnoses and procedures would entail greater complexity or cost for older patients, there was insufficient information for selecting which of these to adjust for, and to what extent, within the Lebanese setting. This remains an important area for future investigations and development of this system. We note that in the 2018 update of the Total Performance Score, the proportion of elderly was captured as a factor, and thus had some influence on determining hospital reimbursement tiers; this was to incentivize hospitals away from avoiding elderly patients.*

*We also acknowledge the importance of capturing comorbidities within casemix index calculation. Unfortunately, the MoPH and hospitals do not routinely code for comorbidities; the field for these is ‘optional’, and as such, any entries were unsuitable for use in this study. The MoPH had agreed in principle that this factor should be included, however there were concerns regarding practical implementation and potential for miss-use. Therefore, this awaits a more comprehensive revision of the coding-reimbursement-auditing mechanisms.*

*Medical cases were divided by length of stay on the basis that they are reimbursed by fee-for-service, and length of stay among these cases has a direct relation to the cost submitted by hospitals for MoPH reimbursement. However, surgical cases are reimbursed on a flat-rate basis, regardless of length of stay. In this investigation we maintained the same categories used by the MoPH to calculate casemix index. However, we acknowledge that development of the system to account for length of stay also among surgical cases would allow a more accurate measurement of case complexity.*

 

- Given that five-year cost averages was used for calculation of CMI, how (if at all) was the inflation and other factors influencing the cost throughout the years taken into account? In other words, was the weight of one particular ICD or procedure code the same throughout the five-year period?

*For surgical cases (flat-rate), the MoPH updated the procedure costs in March 2013, which remained in use until the 2018 update. These updates increased the base-rate of all procedures, to account for inflation. We used weights based on the March 2013 update, also retrospectively up to 2011. For medical cases (fee-for-service), the MoPH undertook a hospital-bed update (base-rate), which is only one component of the bill charged by hospitals to the MoPH, but nevertheless represents an internal accounting for inflation. For medical cases, we used the 5-year average per ICD10 code, without further adjustment.*

 

- It was mentioned in the article that the weight was calculated for each ICD (and procedure code). In table 4 it remains unclear if the separate weights were calculated e.g. for diagnosis code J44.1 and J44. Or was the separate weight calculated for codes J20-J20.9 and J20. However, table 5 which refers to diagnosis codes with the greatest change in terms of CMI, shows different values e.g. for code P22 and P22.0. This is somehow confusing because in general, subcodes of three-character diagnosis code should be clinically and economically meaningful and should have one weight, i.e. codes P22 and P22.0 should be similar in terms of clinical severity and cost. In general (and this really depends on national coding guidelines), if a three-character diagnosis code has subcodes, then the subcode as more specific one should be used instead of three-character code.

*Separate weights were calculated for each code, whether three or four-character. Therefore, J44.1 and J44 had different weights (differing by 14%), as did P22 and P22.0. The decision to have separate weights was based on the review of various codes, which in some cases revealed considerable differences between three and four-character codes. Acknowledging that this is likely due to a mix of factors, including imprecise coding practices and actual diagnosis, but lacking information to resolve these differences, we choose to use weights for each three and four-character code. This approach is similar to that used in the calculation of casemix index by the MoPH. The use of three-character codes is also due for phasing out by the MoPH, which will require four-character codes for all hospitalizations.*
